# Effects of Wheat Naturally Contaminated with *Fusarium* Mycotoxins on Growth Performance and Selected Health Indices of Red Tilapia (*Oreochromis niloticus × O. mossambicus*)

**DOI:** 10.3390/toxins7061929

**Published:** 2015-05-29

**Authors:** Siriporn Tola, Dominique P. Bureau, Jamie M. Hooft, Frederick W. H. Beamish, Michael Sulyok, Rudolf Krska, Pedro Encarnação, Rakpong Petkam

**Affiliations:** 1 Department of Fisheries, Faculty of Agriculture, Khon Kaen University, Khon Kaen 40002, Thailand; E-Mail: pattola4445@gmail.com; 2 UG/OMNR Fish Nutrition Research Laboratory, Department of Animal and Poultry Science, University of Guelph, Guelph, ON N1G 2W1, Canada; E-Mails: dbureau@uoguelph.ca (D.P.B.); jhooft@uoguelph.ca (J.M.H.); 3 Environmental Science, Faculty of Science, Burapha University, Bangsaen, Chonburi 20131, Thailand; E-Mail: billbeamish@hotmail.com; 4 Department IFA-Tulln, University of Natural Resources and Life Sciences, Vienna (BOKU), Konrad-Lorenz-Str. 20, Tulln 3430, Austria; E-Mails: michael.sulyok@boku.ac.at (M.S.); Rudolf.krska@boku.ac.at (R.K.); 5 Biomin Singapore Pte. Ltd., 3791 Jalan Bukit Merah, #08-08, E-Centre@Redhill, Singapore 159471, Singapore; E-Mail: pedro.encarnacao@biomin.net

**Keywords:** mycotoxins, deoxynivalenol, *Fusarium* mycotoxins, red tilapia, growth performance

## Abstract

An 8-week feeding trial was conducted to examine effects of wheat naturally contaminated with *Fusarium* mycotoxins (deoxynivalenol, DON 41 mg·kg^−1^) on growth performance and selected health indices of red tilapia (*Oreochromis niloticus × O. mossambicus*; initial weight = 4.3 g/fish). Five experimental diets were formulated by replacement of clean wheat with naturally contaminated wheat resulting in graded levels of DON and zearalenone (ZEN) (Diet 1 0.07/0.01, Diet 2 0.31/0.09, Diet 3 0.50/0.21, Diet 4 0.92/0.37 and Diet 5 1.15/0.98 mg·kg^−1^). Groups of 50 fish were randomly allocated into each of 20 aquaria and fed to near-satiety for eight weeks. Growth rate, feed intake and feed efficiency of fish fed the experimental diets decreased linearly with increasing levels of *Fusarium* mycotoxins (*p* < 0.05). Although growth depression was associated with feeding diets naturally contaminated with *Fusarium* mycotoxins, especially DON, no biochemical and histopathological parameters measured in blood and liver appeared affected by *Fusarium* mycotoxin concentrations of diets (*p* > 0.05). Though there was no clear evidence of overt DON toxicity to red tilapia, it is recommended that feed ingredients should be screened for *Fusarium* mycotoxin contamination to ensure optimal growth performance.

## 1. Introduction

Feed represents over 60% of operating costs in aquaculture [1]. Efforts to reduce the cost of feed have led to a reduction in the level of costly fishmeal and increased levels of plant ingredients in the feed of most fish species [2,3]. Feeds for tilapia and other omnivorous fish species are currently formulated to contain high levels of plant feedstuffs. Several surveys have found extensive mycotoxin contamination of both plant feedstuffs [4,5,6,7,8]. As a result, fish have a high risk of exposure to feed that might contain significant levels of mycotoxins and this may potentially lead to significant economic losses [8,9,10].

Mycotoxins are secondary toxic metabolites, produced by filamentous fungi, such as *Aspergillus*, *Penicillium* and *Fusarium* fungi. Though numerous mycotoxins commonly occur in feed and feedstuffs, some toxins, namely aflatoxin B1 (AFB1), zearalenone (ZEN), deoxynivalenol (DON), fumonisin B1 (FB1) and ochratoxin A (OTA), draw the most scientific attention due to their toxic potentially adverse impacts on animal health [4,5,7]. Mycotoxin contamination is largely dependent on temperature and moisture conditions and may occur at various stages of production (e.g., in the field, during harvest, transport or storage) [9,11]. *Fusarium* mycotoxins, particularly DON, ZEN, and FB1, are ubiquitous in cereal grains worldwide [7,8,12]. Prevalence of these mycotoxins was found in different geographic regions according to a mycotoxin survey program between 2012 and 2013 in Central Europe, North Europe, North Asia, Africa and South Africa [13]. Notably, in a recent annual survey, 50% to 65% of samples were detected to contain DON concentrations ranging from 0.3 to 1.7 mg·kg^−^^1^ depending on commodity type and geographical region of origin [14].

DON is trichothecene mycotoxins produced mainly by *Fusarium graminearum* and *F. culmorum* that is frequently found in feeds and feedstuffs [5,7]. It acts as a potent inhibitor of protein synthesis via binding to the 60S ribosomal subunit and interfering with peptidyl transferase activity. The 9,10 double bond and the C-12,13 epoxide are involved in inhibition of protein synthesis during the elongation and termination steps [15,16]. DON causes deleterious effects in animals. Clinical symptoms of acute toxicity (e.g., vomiting and diarrhea) and symptoms of sub-chronic and chronic toxicity e.g., decreased growth rate, feed intake, feed efficiency and histopathological alteration in organs of animals exposed to DON have been reported [17,18]. Influence on serotoninergic activity is one mechanism of action that leads to vomiting and feed refusal after exposure to high and low levels of DON, respectively [19,20]. Immunosuppression resulting in decreased disease resistance is also believed to be associated with DON [21,22].

The effects of DON on animals vary depending on the nutritional and health status of animals prior to exposure, environmental conditions, and forms of DON (e.g., purified and natural forms) as well as dose and duration of exposure [23,24]. Pigs are considered as most sensitive animal species to DON; concentrations as low as 1 to 2 mg·kg^−1^ caused decreases in growth and feed intake following oral exposure. Pigs exhibit feed refusal and vomiting when fed diets containing 12 and 20 mg DON kg^−1^, respectively [25,26]. In contrast, the growth performance of poultry and ruminants were not found to be significantly affected by consumption of diets containing 9.7 and 8.5 mg DON kg^−1^, respectively [27,28]. Only few studies have reported adverse impacts of dietary DON on aquatic species, e.g., rainbow trout (*Oncorhynchus mykiss*) and channel catfish (*Ictalurus punctatus*) [24,29]. Significant reduction in growth, feed efficiency and feed intake were observed in rainbow trout fed diets containing corn artificially contaminated with graded levels of DON ranging from 1 to 12.9 mg·kg^−1^ for eight weeks [29]. More recently, Hooft, *et al.* [24] demonstrated that rainbow trout are extremely sensitive to low dietary levels of DON (<1 mg·kg^−1^). These authors observed significant linear or quadratic decreases in weight gain, feed intake, feed efficiency, retained nitrogen, recovered energy, energy retention efficiency and nitrogen retention efficiency of rainbow trout fed diets containing low levels of DON ranging from 0.3 to 2.6 mg·kg^−1^ from naturally contaminated corn. In addition, livers of rainbow trout fed dietary DON exhibited fatty infiltration and pyknosis and karyolysis were observed in hepatocytes [24]. Conversely, channel catfish appeared to be more resistant, as diets with high levels of dietary DON (10 mg·kg^−1^) did not affect performance [21,30].

It is apparent that adverse impacts on growth performance and histopathological alteration of organs associated with feeding dietary DON vary considerably among fish species. Additionally, some evidence has shown co-occurrence of several *Fusarium* metabolites in naturally contaminated feeds and grains [31,32,33,34]. To our knowledge, the effects of feeding diets containing DON on tilapia, an economically important hybrid fish in South East Asia [1], have not been investigated. In the present study, wheat naturally contaminated with *Fusarium* mycotoxins (DON 41 mg·kg^−1^) was used as a source of DON to examine effects of diets containing graded levels of DON from naturally contaminated wheat on growth performance, selected health parameters and histopathological alteration of red tilapia.

## 2. Results and Discussion

### 2.1. Chemical Composition of Experimental Diets

All experimental diets were formulated to be isonitrogenous and isoenergetic and to meet all nutrient requirements of tilapia as estimated by the NRC [35]. There was no significant difference (*p* > 0.05) nutritional composition among diets.

### 2.2. Determination of Mycotoxins and Fungal Metabolites in Diets

The replacement of clean wheat with naturally contaminated wheat resulted in five diets, containing graded levels of DON and ZEN. The concentrations of DON and ZEN as analyzed by using liquid chromatography—tandem mass spectrometry (LC-MS/MS) in Diets 1–5 were 0.07/0.01, 0.31/0.09, 0.50/0.21, 0.92/0.37 and 1.15/0.98 mg·kg^−1^, respectively. A total of 50 secondary fungal metabolites were determined by LC-MS/MS. Surprisingly, some *Fusarium* metabolites, including aurofusarin (0.01 to 2.46 mg·kg^−1^), culmorin (0.20 to 1.39 mg·kg^−1^) and 15-hydroxyculmorin (0.06 to 1.83 mg·kg^−1^), were detected in all diets at higher levels than DON in this study.

The co-occurrence of fungal metabolites in feed and feed ingredients naturally contaminated with mycotoxins has been shown in several reports. The findings in the present study are in agreement with previous reports [4,5,8,31,32,33] indicating that use of grains naturally contaminated with *Fusarium* mycotoxins results in variations in the concentrations of *Fusarium* mycotoxins (e.g., DON and ZEN), as well as in inescapable fungal metabolites, namely *Fusarium* metabolites and *Alternaria* metabolites (e.g., alternariol, AOH; alternariolmonomethyl ether, AME).

### 2.3. Growth Performance

Growth performance (e.g., weight gain, thermal-unit growth coefficient, feed intake and feed efficiency) decreased linearly with increasing levels of *Fusarium* metabolites ingested (Table 1). Some evidence suggests that intake of a combination of mycotoxins could result in a synergistically/additively adverse impact on animals than that of a single mycotoxin [36,37,38]. The depression of growth in red tilapia in the present study may be related to presence of any one of these *Fusarium* metabolites, or a possible interaction among them. This remains to be investigated. However, the current data on toxic effects of these fungal metabolites on animals is insufficient to decisively clarify whether they could impair growth of red tilapia in the present study. Sauer, *et al.* [39] demonstrated that a diet containing high levels of AME 24 (mg·kg^−1^) and AOH (39 mg·kg^−1^) for 21 days did not reduce weight gain or alter tissues on gross and microscopic examinations. In addition, Alexander, *et al.* [40] suggested that AOH and AME are less toxic to animals than any other in the *Alternaria* group. Rotter, *et al.* [41] reported that diets containing 2 mg culmorin kg^−1^ alone or in combination with 6 mg DON for 21 days did not reduce weight gain and feed intake of pigs (average weight = 22.8 kg/each). Based on these findings, trace amounts of AOH (0.12 mg·kg^−1^), AME (0.15 mg·kg^−1^) and culmorin (1.39 mg·kg^−1^) in the present study likely did not cause any deleterious effects on the growth of red tilapia. Furthermore, a small amount of ZEN (up to 0.4 mg·kg^−1^) was detected in the experimental diets in the study of Hooft, *et al.* [24] and 4 mg·kg^−1^ was detected in corn used as a source of dietary DON in the study of Woodward, *et al.* [29]. Although there was evidence suggesting growth promoting action in ruminants and assessment of oestrogenic potency *in vitro* [42,43], possible additive or synergistic effects on fish growth resulting from a combination of DON and ZEN require further investigation [5,44,45].

In red tilapia, weight gain, growth rate (expressed as thermal-unit growth coefficient, TGC), feed intake and feed efficiency decreased linearly (*p* < 0.01) with increasing levels of DON ranging from 0.07 to 1.15 mg·kg^−1^. Additionally, diets containing increasing levels of DON were associated with linear and quadratic decreases in mortality (*p* < 0.05). Growth performance of red tilapia in the present study was in agreement with previous studies on rainbow trout in which growth rate and feed intake were linearly depressed by increasing levels of dietary DON [24,29].

**Table 1:** Effects of diets naturally contaminated with *Fusarium* mycotoxins on growth performance and mortality rate of red tilapia (initial average weight = 4.3 g/fish) over the 8-week trial. Mortality is based on number of deaths after the 8-week trial relative to initial number.

Diet	DON	WG ^a^	TGC ^b^	FI ^c^	FE ^d^	Mortality
	(mg·kg^−1^)	(g/Fish)		(g/Fish)	(Gain/Feed)	(%)
**1** (0.0% ^e^)	0.07	37.4	0.108	52.5	0.71	12
**2** (1.2%)	0.31	34.9	0.104	49.5	0.70	14
**3** (2.5%)	0.50	34.0	0.102	49.5	0.69	17
**4** (5.0%)	0.92	31.6	0.099	47.2	0.67	10
**5** (10.0%)	1.15	29.6	0.095	45.8	0.65	6
**Significance ^f^**						
**Linear**		*p* < 0.01	*p* < 0.01	*p* < 0.01	*p* < 0.01	*p* < 0.05
**Quadratic**		N.S. ^g^	N.S.	N.S.	N.S.	*p* < 0.05
**S.E.M ^h^**		1.3	0.002	1.3	0.01	2.2

Note: *n* = 4 for each treatment; ^a^ Weight gain; ^b^ Thermal-unit growth coefficient; ^c^ Feed intake; ^d^ Feed efficiency; ^e^ Proportion of contaminated wheat in experimental diet; ^f^ Significance of the orthogonal linear and quadratic contrasts; ^g^ Not statistically significant (*p* > 0.05); ^h^ S.E.M. = Standard error of mean.

In rainbow trout, feeding dietary DON as low as 1 mg·kg^−1^ caused a reduction in feed intake, feed efficiency and growth rate [24,29]. Woodward, *et al.* [29] reported that significant reductions in feed intake, feed efficiency and weight gain were associated with increasing levels of DON ranging from 1 to 12.9 mg·kg^−1^ diet. Hooft, *et al.* [24] found that feeding naturally contaminated diets containing DON ranging from 0.3 to 2.6 mg·kg^−1^ diet resulted in significant linear or quadratic reductions in feed intake feed efficiency and growth rate (TGC) of rainbow trout. Observed differences in sensitivity of rainbow trout to DON in these two studies could be accounted for, in part, by the use of different sources of DON. Woodward, *et al.* [29] used artificially infected corn while Hooft, *et al.* [24] used naturally contaminated corn. Additionally, the initial weight of fish used in the study of Hooft, *et al.* [24] was half that in the study by Woodward *et al.* [29] (23 and 50 g/fish). In contrast, feed intake and weight gain in channel catfish were not affected by graded levels of DON in either naturally contaminated wheat and corn, or purified DON ranging from 0 to 10 mg DON kg^−1^ diet [21,30]. However, catfish fed diet containing >15.0 mg DON kg^−1^ diet from naturally contaminated sources of wheat exhibited reduced growth and poor feed conversion ratio [30]. Clearly, channel catfish are more tolerant to DON than rainbow trout and red tilapia demonstrating differences among species.

Fishes vary in their sensitivity to DON which may be associated with differences inthe detoxification capacity of microorganisms in the digestive tract and liver to transform DON to the less toxic metabolites (e.g., de-epoxy deoxynivalenol, DOM-1; DON-3-glucuronide) [46,47,48]. DON is transformed to de-epoxy deoxynivalenol (DOM-1) via deepoxidation and deacetylation by microorganisms from the digestive tract [46,47]. In common carp, *Cyprinus carpio*, hepatic microsomes in the liver have been shown to transform DON to DON-3-glucuronide [46]. Microbes from the digestive tract of brown bullhead (*Ameiurus nebulosus*) were more capable of transforming DON to DOM-1 than brown trout (*Salmo trutta*), pink salmon (*Oncorhynchus gorbuscha*) and other fishes [48].

The significant reduction in feed intake by red tilapia in the present study is in agreement with previous findings in rainbow trout and pigs. Reduced intake of feed contaminated with *Fusarium* mycotoxins (e.g., DON, T-2 toxin, fusaric acid) might be a response to gastrointestinal tract irritation by DON and co-occurring mycotoxins [44,45] inhibition of gastric emptying [49] and enhanced production serotonin (5-hydroxytryptamine, 5-HT), a brain neurotransmitter [20], linked to behavioral changes such as loss of appetite, vomiting, and emesis [49]. Nevertheless, Hooft, *et al.* [24] showed that rainbow trout pair-fed a diet containing 0.3 mg DON kg^−1^ had higher growth rates, feed efficiency, and carcass crude protein content than those fed with a higher concentration of DON (2.6 mg·kg^−1^). This suggests dietary DON does not simply result in a reduction in feed intake of rainbow trout but also negatively affects nutrient metabolism [24]. Accordingly, Lun, *et al.* [50] reported a depression of growth of pig associated with dietary DON may be related to the disturbance of minerals absorption and/or metabolism.

Significant linear and quadratic decreases (*p* < 0.05) in the mortality of red tilapia were associated with feeding diets containing increasing levels of DON in the present study. Manning, *et al.* [21] did not observe mortality of channel catfish fed diets containing high levels of DON (10 mg·kg^−1^) from naturally contaminated corn. Likewise, no significant difference in mortality of rainbow trout fed a diet containing 6.4 mg·kg^−1^ purified DON or fish pair-fed the control diet was observed by Ryerse, *et al.* [51]. Interestingly, Manning, *et al.* [21] demonstrated that feeding diets containing 5 and 10 mg DON kg^−1^ significantly decreased mortality of channel catfish during a trial of 21-day post-challenge with the pathogenic bacterium (*Edwardsiella ictaluri*). This finding was supported by Ryerse, *et al.* [52], who observed a significant reductions in the cumulative mortality of rainbow trout fed diets containing 4.1 or 5.9 mg·kg^−1^ DON from naturally contaminated corn compared to control and pair-fed groups following infection with *Flavobacterium psychrophilum*. Likewise, feeding a diet containing 6.4 mg·kg^−1^ purified DON significantly reduced mortality of rainbow trout after experimental *F. psychrophilum* infection compared to the control group (<0.1 mg·kg^−1^ DON) [51]. The authors suggest that reduced feed intake associated with DON may positively influence survival during disease outbreaks [51]. The exact cause of the mortalities observed in the present study is unclear.

### 2.4. Hematological and Biochemical Parameters

Hematocrit (Hct), plasma alanine aminotransferase (ALT), plasma aspartate aminotransferase (AST) and hepatosomatic index (HSI) of fish were not significantly affected by the experimental diets (Table 2). Hepatosomatic index (HSI) in tilapia fed the control and DON-treated diets were similar which suggests that dietary DON did not cause extensive changes to their liver.

However, there were trends of the decreases in both Hct and HSI with increased ingestion of dietary DON. In accord, Hct in channel catfish and rainbow trout did not exhibit a significant change when feeding 17.5 and 1.96 mg DON kg^−1^ diet, respectively [30,53]. Further, Matejova, *et al.* [53] reported that AST and ALT in rainbow trout did not differ significantly from those for a control group when fed diets artificially contaminated with 1.96 mg DON kg^−1^.

**Table 2:** Effects of diets naturally contaminated with *Fusarium* mycotoxins on hematological and biochemical parameters, and hepatosomatic index of red tilapia (initial average weight = 4.3 g/fish) over the 8-week trial.

Diet	DON ^a^	AST ^b^	ALT ^c^	Hct ^d^	HSI ^e^
	(mg·kg^−1^)	IU/L	IU/L	(%)	(%)
**1** (0.0% ^f^)	0.07	592	96	34	2.40
**2** (1.2%)	0.31	477	69	32	2.18
**3** (2.5%)	0.50	574	103	33	1.94
**4** (5.0%)	0.92	350	90	32	1.87
**5** (10.0%)	1.15	527	146	31	1.97
**Significance ^g^**					
**Linear**		N.S. ^h^	N.S.	N.S.	N.S.
**Quadratic**		N.S.	N.S.	N.S.	N.S.
**S.E.M. ^i^**		142	38	1.5	0.18

Note: *n* = 12, (12 individual fish per treatment were used for analysis); ^a^ Deoxynivalenol; ^b^ Plasma aspartate aminotransferase; ^c^ Plasma alanine aminotransferase; ^d^ Hematocrit; ^e^ Hepatosomatic Index; ^f^ Proportion of contaminated wheat in experimental diet; ^g^ Significance = significance of the orthogonal linear and quadratic contrasts; ^h^ Not statistically significant (*p* > 0.05); ^i^ S.E.M. = Standard error of mean.

### 2.5. Histopathological alterations in livers

**Figure 1:**
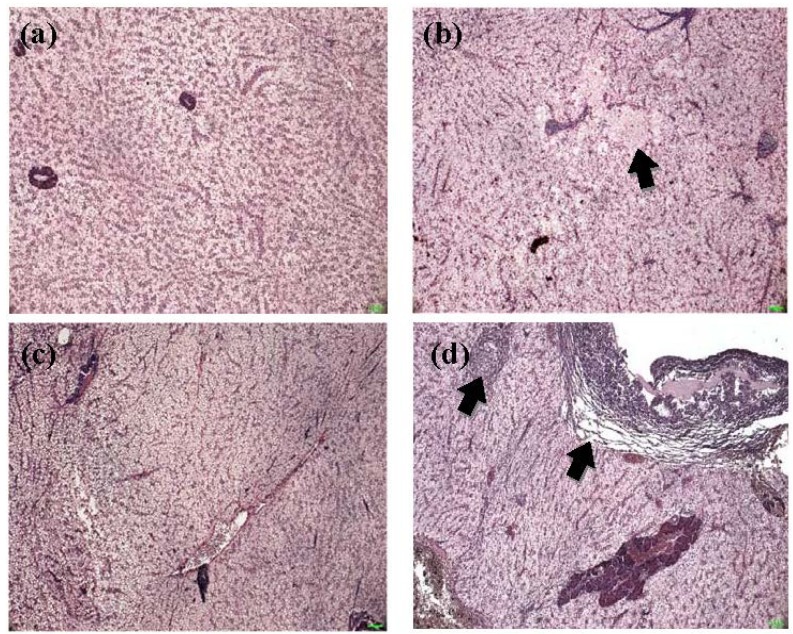
Light microscopy (×250, H & E stain) of the liver of red tilapia fed experimental diets for eight weeks. (**a**) Liver of red tilapia fed control diet (0.07 mg DON kg^−1^); (**b**) distribution of focal necrosis (arrow) in liver of red tilapia fed Diet 3 (0.50 mg DON kg^−1^); (**c**) cytoplasmic vacuolation in liver of red tilapia fed Diet 2 (0.31 mg DON kg^−1^) and (**d**) subcapsular edema (arrow) in liver of red tilapia fed Diet 2 (0.31 mg DON kg^−1^).

Although histopathological change is not a toxicity-specific response, hepatocytes are known to respond to a variety of toxicants [54]. Most red tilapia fed the control and other diets had healthy livers (*n* = 12), only few livers appeared to have changes upon gross and microscopic examinations (Figure 1 (a)). Areas of focal necrosis were observed in red tilapia fed diet 3 (Figure 1 (b)) and 5. Cytoplasmic vacuolation was observed in each liver from fish fed diet 2 (Figure 1 (c)) and 3. Seven samples of fish fed diets 2 (Figure 1 (d)), 3 and 4 showed subcapsular edema in both of the gross and microscopic examinations.

Histopathological alterationsin red tilapia were not associated with DON dose, based on the scoring system by Bernet *et al.* [55], although lesions were observed in hepatic tissue in some fish. Histopathological alterations in livers and kidneys of rainbow trout have been reported and related to dietary DON [24,53]. Subcapsular edema were observed in livers of rainbow trout fed 1.4 mg DON·kg^−1^ along with fatty infiltration and phonotypical alteration of rainbow trout fed 2.6 mg DON kg^−1^ [24]. Kidneys of rainbow trout displayed severe hyaline droplet denegation in tubular epithelial cells when fed 1.96 mg DON·kg^−1^ [53]. Focal necrosis and subcapsular edema observed in hepatic tissues of red tilapia were similar to the findings of Hooft *et al.* [24], who suggested edema may be associated with the inhibition of protein synthesis by DON resulting in a lack of plasma protein. Furthermore, cytoplasmic vacuolation was noticed in some livers of red tilapia fed dietary DON, perhaps a result of lipid accumulation or lack of glycogen in hepatocytes. Liver is considered as a good indicator of nutritional and toxic pathology because of its function in metabolizing substances from the digestive tract [56].

## 3. Experimental Section

### 3.1. Experimental Condition

This experiment was conducted at Applied Centre of Aquaculture Nutrition (ACAN), Biomin (Thailand) Co., Ltd. (Bangkok, Thailand). Twenty of 120-L aquaria in a recirculation indoor system with a flow rate of approximately 1.5 cm^3^ per second flow rate per tank were used. Each tank was continuously aerated to assure ambient dissolved concentrations near air saturation. The average values of each water quality parameter over eight weeks were: dissolved oxygen = 4.44 ± 0.59 mg·L^−1^, temperature = 30.50 ± 0.6 °C, pH = 6.95 ± 0.16, ammonia = 0.42 ± 0.12 mg·L^−1^, nitrate = 20 mg·L^−1^, and nitrite = 0.40 ± 0.13 mg·L^−1^. Feces and sediment were removed every morning before feeding. Water volume (50%) in the recirculation system was replaced with dechlorinated tap water weekly.

### 3.2. Experimental Animal

Two thousand juvenile red tilapia (*O. niloticus × O. mossambicus*; initial average weight = 4.3 g/fish) obtained from a commercial farm (Bangkok, Thailand) were held in aquaria for three weeks. Prior to the beginning of an experiment, 50 fish of similar body weight were weighed at the onset of an experiment and randomly distributed into each of 20 aquaria. Fish were fed to near-satiety with the experimental diets three times daily for eight weeks (900, 1300 and 1600 h on the workday, and once per day at 900 h on the weekend). Mortality was recorded daily and feed intake was recorded weekly.

### 3.3. Experimental Diets

Naturally contaminated wheat (41 mg DON kg^−^^1^) and clean wheat flour (0.23 mg DON kg^−^^1^) were used in the dietary preparation and referred to as “contaminated wheat” and “clean wheat”, respectively. Five isonitrogenous and isoenergetic diets containing graded levels of DON were formulated by replacement of clean wheat with contaminated wheat (Table 3). Spaghetti-like pellets, 2 mm diameter, were made using a meat grinder and were broken by hand to the appropriate size for fish. The pellets were dried in an oven at 70 °C for two hours, left to cool overnight and kept in plastic bags until used. Five hundred grams of each experimental diet were sampled and ground for proximate analysis according to AOAC [57] and mycotoxin analysis by LC-MS/MS.

**Table 3:** Ingredient composition of the five isonitrogenous and isoenergetic diets and levels of detected *Fusarium* mycotoxins.

Ingredients	Experimental Diets				
(100%)	1	2	3	4	5
Cassava	15.0	15.0	15.0	15.0	15.0
Soybean meal	25.0	25.0	25.0	25.0	25.0
Clean wheat ^a^	19.6	18.4	17.1	14.6	9.6
Contaminated wheat ^b^	-	1.2	2.5	5.0	10.0
Rice bran	20.0	20.0	20.0	20.0	20.0
Fish meal ^c^	15.0	15.0	15.0	15.0	15.0
Vitamin premix ^d^	0.4	0.4	0.4	0.4	0.4
Fish oil	2.0	2.0	2.0	2.0	2.0
Soybean oil	3.0	3.0	3.0	3.0	3.0
Total	100	100	100	100	100
**Proximate analysis (% dry matter basis)**					
Dry matter (%)	94.4	95.6	94.9	95.5	94.6
Crude protein (%)	25.1	25.2	24.3	24.5	24.1
Lipid (%)	6.7	6.7	6.9	6.9	7.0
Ash (%)	8.5	8.5	8.5	8.5	8.6
Gross energy (kJ/g)	17.8	17.9	17.9	17.8	17.8
***Fusarium* mycotoxins (mg·kg^−1^ diet)**					
Deoxynivalenol (DON)	0.07	0.31	0.50	0.92	1.15
Zearalenone (ZEN)	0.01	0.09	0.21	0.37	0.98
**Other *Fusarium* metabolites (mg·kg^−1^ diet)**					
Aurofusarin	0.01	0.30	0.66	1.28	2.46
Rubrofusarin	0.02	0.05	0.12	0.23	0.49
Culmorin	0.02	0.18	0.26	0.50	1.39
15-Hydroxyculmorin	0.06	0.24	0.37	0.67	1.83
***Alternaria* metabolites (mg·kg^−1^ diet)**					
Alternariol	0.001	0.01	0.02	0.04	0.12
Alternariolmonomethyle-ther	0.01	0.02	0.04	0.06	0.14

Note: ^a^ Clean wheat flour contains 0.23 mg DON kg^−1^ analyzed by LC-MS/MS; ^b^ Wheat naturally contaminated with 41 mg DON kg^−1^; ^c^ Contains >60% crude protein; ^d^ Vitamin premix (Premix BA 193C, Biomin) provides per kg diet: vitamin A, 2.000.000 IU; vitamin D3, 480.000 IU; vitamin E, 20 g; vitamin C, 10 g; vitamin K3, 3 g; vitamin B1, 2.5 g; vitamin B2, 3 g; vitamin B6, 3.5 mg; vitamin B12, 0.01 g; niacine, 5 g; panthotenic acid, 5 g; choline 60%; Zn, 70 g; Fe, 14 g; Cu 2.4 g; Mn, 12 g; Co, 0.025 g; I, 1 g; Se 0.12 g; folic acid, 1.7 g; Biotin, 0.05 g; inositol, 10 g.

### 3.4. Mycotoxin Analysis of Dietary Samples

LC-MS/MS was used to analyze for multiple mycotoxins and fungal metabolites [58], 5 g of each sample were extracted for 90 min with 20 mL of acetonitrile/water/acetic acid (79:20:1, *v/v/v*) on a rotary shaker (GFL 3017, GFL, Burgwedel, Germany). The crude extracts were diluted 1 + 1 (*v + v*) with acetonitrile/water/acetic acid (20:79:1, *v/v/v*) and 5 µL of the diluted extract were injected.

Detection and quantification were performed with a liquid chromatography/tandem mass spectrometry (LC-MS/MS) system (QTrap5500, Applied Biosystems, Foster City, CA, USA) equipped with an electrospray ionisation (ESI) source (TurboIonSpray) and an ultra-high performance liquid chromatography system (1290 Series, Agilent Technologies, Waldbronn, Germany). Chromatographic separation was performed at 25 °C on a Gemini C18-column, 150 × 4.6 mm inner diameter, (5 μm particle size), equipped with a C18 security guard cartridge, 4 × 3 mm i.d. (all from Phenomenex, Torrance, CA, USA). Elution was carried out in binary gradient mode. Both mobile phases contained 5 mM ammonium acetate and were composed of methanol/water/acetic acid 10:89:1 (*v/v/v*; eluent A) and 97:2:1 (*v/v/v*; eluent B), respectively. After an initial time of 2 min at 100% A, the proportion of B was increased linearly to 50% within 3 min. Further linear increase of B to 100% within 9 min was followed by a hold-time of 4 min at 100% B and 2.5 min column re-equilibration at 100% A. The flow rate was 1000 μL/min. Data acquisition was performed in the scheduled multiple reaction monitoring (sMRM) mode both in positive and negative polarities in two separate chromatographic runs. The sMRM detection window of each analyte was set to the respective retention time ±27 s and ±42 s in positive and negative modes, respectively. The target scan time was set to 1 s. Confirmation of positive analyte identification was obtained by the acquisition of two sMRMs per analyte (with the exception of moniliformin and 3-nitropropionic acid that each exhibited only one fragment ion), which yields 4.0 identification points according to commission decision 2002/657/EC (EU 2002). Analyst^®^ software version 1.5.1 (AB Sciex, Foster City, CA, USA) was used to control the LC-MS/MS instrument, as well as for automatic and manual integration of the peak. Quantification of the >300 metabolites included in the method was done based on linear, 1/*x* weighed calibration curves derived from the analysis of serial dilutions of a multi-analyte stock solution. Results were not corrected for apparent recoveries due to lack of a suitable blank sample. The accuracy of the method was verified on a routine basis by participation in a proficiency testing scheme organized by BIPEA (Gennevilliers, France), which included samples of animal feed. The following analytes were positively identified in one or more of the samples: DON, DON-3-Glucoside, ZEN, zearalenon-4-Sulfat, alpha zearalenol, beta zearalenol, sterigmatocystin, averufin, 3-nitropropionic acid, moniliformin, siccanol, equisetin, apicidin, enniatin B, enniatin B1, enniatin A1, enniatin A, beauvericin, monocerin, aurofusarin, rubrofusarin, culmorin, 15-hydroxyculmorin, tenuazonic acid, alternariol, alternariolmethylether, macrosporin, tentoxin, secalonic acid D, chanoclavin, curvularin, tryptophol, brevinamid F, emodin, chrysophanol, ergometrin, ergometrin, ergosinin, ergosin, ergocornin, ergocorninin, ergotamines, ergocryptin, ergocryptinin, ergocristin, ergocristinin, linamarin, and lotaustralin, while all other investigated metabolites were below their respective limits of detection.

### 3.5. Sample Collection

All fish from each aquarium were weighed for initial and final total body weight as well as at the end of 4th and 8th-week. Feed intake was recorded weekly. At the end of the experiment, three fish from each aquarium were anaesthetized with eugenol (Sigma-Aldrich Ptd., Ltd., Singapore, Singapore) prior to blood collection. Blood samples were analyzed for Hct, ALT and AST. Livers were dissected for HSI, and histological examination were calculated and conducted, respectively.

### 3.6. Laboratory Analysis

AST and ALT in plasma were examined using Architect c 16000Analyzer (Abbott Laboratories, Abbott Park, IL, USA). Hct was estimated using a micro HT centrifuge (DSC-100MH-2 Digisystem Laboratory Instrument Inc., Taipei, Taiwan). Crude protein, ether extract (lipid), ash, moisture, dry matter and gross energy of diets were analyzed. Samples of experimental diets were analyzed for crude protein (CP), ether extract (EE, lipid), ash, moisture, dry matter (DM) and gross energy (GE) according to [57]. Tecator Kjeltec digestion and distillation units were used for CP analysis and the percentage of total nitrogen was determined based on a dry matter basis (%N × 6.25). Lipid was extracted from samples using petroleum ether by Soxhlet apparatus and GE was analyzed using automated bomb calorimeter (AC500 Isoperibol bomb calorimeter, LECO Corporation, St. Joseph, MI, USA).

### 3.7. Histopathological Examination

Sample tissues were processed according to Clark [59]. In brief, tissues were processed using a Shandon citadel 2000 tissue processor (International medical equipment Inc., San Marcos, CA, USA). Then they were dehydrated, cleared, infiltrated with liquid paraffin, and finally embedded in paraffin blocks.

Tissues were sectioned at 4 µm thick using a Leitz 1512 rotary microtome and stained with Hematoxylin and Eosin (H & E stain). Mounted slides were observed under a light microscope. Five liver specimens of fish from the lowest and highest DON level treatments were inspected. Images were captured by a digital camera (Micropublisher 5.0, Qimage, BC, Canada) coupled with a light microscope (Openlab) at the UG/OMNR Fish Nutrition Research Laboratory, Department of Animal and Poultry Science, University of Guelph (Guelph, ON, Canada).

### 3.8. Calculations and Statistical Analysis

Thermal-unit growth coefficient (TGC) was expressed as growth rate and calculated for each aquarium as: [100 × (FBW1/3 − IBW1/3)/Σ(Temp (°C) × number of days)], where: FBW = final body weight (g/fish); IBW = initial body weight (g/fish). Feed efficiency (FE) was calculated as live body weight gain/dried feed intake (FI), where: FI = total dry feed/number of fish; live body weight gain = (FBW/final number of fish) − (IBW/initial number of fish). HSI was calculated as HSI = (liver weight (g)/FBW × 100). Mortality rate (%) was calculated as percentage of mortality (%) = initial number of fish − final number of fish × 100)/initial fish number).

All parameters, such as body weight gain, TGC, feed intake, feed efficiency, mortality rate, HSI, Hct, AST and ALT are presented as treatment mean and standard error of the mean (SEM). The GLM procedure of SAS for Windows (SAS version 9.2, SAS Institute Inc., Cary, NC, USA) was used to perform linear and quadratic orthogonal polynomial contrasts. Statistical significance was declared at *p* < 0.05.

## 4. Conclusions

Impairment of growth performance of red tilapia found in the present study is associated with diets contaminated with *Fusarium* mycotoxins, particularly DON. The adverse impacts observed in the present study may be a possible result of DON contamination alone or due to synergistic effects resulting from co-occurrence of DON and other *Fusarium* metabolites, which are commonly found in naturally contaminated feed. The effects of these *Fusarium* metabolites on the growth of animals are still largely unknown. Hence, the effects of consumption of multiple mycotoxins on animals and fish require further investigation. Inclusion of plant ingredients naturally contaminated with *Fusarium* mycotoxins in fish feed not only increases the risk of exposing farmed fish to DON but may also result in contamination of fish feeds with unidentified *Fusarium* metabolites. Consequently, it is necessary to screen for *Fusarium* mycotoxins and other fungal metabolites in feed and feed ingredients before use in order to ensure good growth performance.
